# Optimization of *in planta* methodology for genome editing and transformation in *Citrus*

**DOI:** 10.3389/fpls.2024.1438031

**Published:** 2024-07-12

**Authors:** Archana Khadgi, Cintia H. D. Sagawa, Corina Vernon, Benoit Mermaz, Vivian F. Irish

**Affiliations:** ^1^ Department of Molecular, Cellular, and Developmental Biology, Yale University, New Haven, CT, United States; ^2^ Department of Ecology and Evolutionary Biology, Yale University, New Haven, CT, United States

**Keywords:** citrus transformation, *in planta* transformation, tissue-culture free transformation, CRISPR/Cas9, sweet genes

## Abstract

Genetic transformation of many plant species relies on *in vitro* tissue culture-based approaches. This can be a labor-intensive process, requiring aseptic conditions and regenerating often recalcitrant species from tissue culture. Here, we have optimized an *in planta* transformation protocol to rapidly transform commercial citrus cultivars, bypassing the need for tissue culture. As a proof of concept, we used *in planta* transformation to introduce CRISPR/Cas9 constructs into Limoneira 8A Lisbon lemon and Pineapple sweet orange, cultivars that are challenging to transform with conventional techniques. Using our optimized protocol, the regeneration rate was significantly increased from 4.8% to over 95%, resulting in multiple gene-edited lines in lemon. We also successfully recovered gene-edited Pineapple sweet orange lines using this protocol; the transformation efficiency for these cultivars ranged between 0.63% and 4.17%. Remarkably, these lines were obtained within three months, making this *in planta* protocol a rapid methodology to obtain transformed citrus plants. This approach can rapidly and effectively introduce key genetic changes into a wide variety of citrus cultivars.

## 1 Introduction

Citrus is the most extensively produced tree fruit crop in the world and there have been constant efforts in improving existing varieties. Traditional breeding approaches in this genus are difficult and time-consuming due to their prolonged juvenile period, high heterozygosity, and complex reproductive biology (Talon and Gmitter, 2008). Genetic engineering technologies offer an alternative approach to conventional breeding programs (Cuenca et al., 2018; Conti et al., 2021). Genetic manipulation using traditional plant transformation methods, including both *Agrobacterium*-mediated transformation or direct DNA delivery through biolistic bombardment, generally relies on *in vitro* tissue culture to generate transformed plants. This process can be labor-intensive, requiring aseptic conditions and the ability to regenerate whole plants from excised tissues. Many citrus varieties are difficult to regenerate, precluding the use of transformation to engineer such varieties. Although optimization of tissue culture approaches have improved the recovery of transgenic citrus varieties in some cases (Dominguez et al., 2022), developing a transformation protocol that excludes the use of tissue culture can be invaluable.

*In planta* transformation is a tissue culture-independent approach to obtain transformed plants. It was first routinely used in *Arabidopsis* in 1987 (Feldmann and David Marks, 1987). Since then, *in planta* transformation has been exploited in *Arabidopsis* using different infection techniques, including imbibition of seed (Feldmann and David Marks, 1987), floral dip (Clough and Bent, 1998), and vacuum infiltration (Bechtold and Pelletier, 1998). Successful transgenesis using *in planta* transformation has already been achieved in many plant species such as jatropha, wheat, soybean, cotton, pigeon pea, tomato, rice, maize and jute (Hu and Wang, 1999; Chumakov et al., 2006; Keshamma et al., 2008; Sajib et al., 2008; Sankara Rao et al., 2008; Agarwal et al., 2009; Yasmeen et al., 2009; Jaganath et al., 2014). Developing citrus transformation via an *in planta* approach would allow for many more laboratories to generate gene-edited varieties aimed at circumventing disease, improving fruit quality or other important traits.

*In vitro* citrus transformation is a long process, taking approximately six months to generate a plantlet, and often requires micrografting of transformed explants onto vigorous rootstocks (Conti et al., 2021). Moreover, genetic transformation in citrus currently relies on *in vitro* tissue culture approaches, requiring sterile conditions and intensive labor, particularly due to the difficulty of regenerating most citrus cultivars (Hao and Deng, 2002; Bairu et al., 2011). In pomelo (*Citrus maxima*), an *in planta* transformation protocol has been successfully utilized to obtain transgenic plants (Zhang et al., 2017a). A similar protocol has been tested in Valencia sweet orange with a transformation efficiency varying between 17.82% to 25.42% between different methods (Xie et al., 2020).

Here, we optimize an *in planta* transformation protocol in Limoneira 8A Lisbon lemon (*Citrus limon* L. Burm.f.), hereafter termed lemon. This protocol was also tested in Pineapple sweet orange (*Citrus sinensis* L. Osbeck), Madam Vinous sweet orange (*Citrus sinensis* (L.) Osbeck), Carrizo citrange (*Citrus sinensis* ‘Washington’ sweet orange X *Poncirus trifoliata*) and Swingle citrumelo (*Citrus paradisi* Macfadyen X *Poncirus trifoliata*) citrus cultivars. We examined various factors that could potentially affect the regeneration and transformation efficiency in these cultivars. For the test constructs used in these transformation studies, we employed constructs targeting three *Sugars Will Eventually be Exported Transporter* (*SWEET*) genes: *SWEET10*, *SWEET12*, and *SWEET15*, utilizing various combinations for CRISPR/Cas9 gene editing. As a proof of concept, we simultaneously mutated *SWEET10*, *SWEET12* and *SWEET15* in lemon and in Pineapple sweet orange using this method. By establishing an efficient *in planta* transformation system, citrus transformation can become faster, more cost-effective, and circumvent the drawbacks associated with *in vitro* transformation methods.

## 2 Materials and methods

### 2.1 Plant materials

The seed coats of Limoneira 8A Lisbon lemon (University of California, Riverside) were peeled, and 25-30 seeds were germinated in Promix soil in a 5” round pot. The pots were covered with Saran wrap and placed in the dark at 28°C for 7-10 days. The soil was initially well-watered and subsequently watered every three to five days. After germination, the seedlings were exposed to light at 28°C and grown for two to three weeks. Similarly, Pineapple sweet orange (*Citrus sinensis* L. Osbeck), Madam Vinous sweet orange (*Citrus sinensis* (L.) Osbeck), Carrizo citrange *Citrus sinensis* ‘Washington’ sweet orange X *Poncirus trifoliata*) and Swingle citrumelo (*Citrus paradisi* Macfadyen X *Poncirus trifoliata*) were also germinated following the same procedure.

For the *in planta* experiment conducted in glass tubes, two seeds per glass tube, with seed coat peeled, were germinated *in vitro* (MS with vitamins, 30 g/L sucrose, 2.5 g/L Phytagel, pH 5.8) in the dark at 24°C for 3-4 weeks. The seedlings were grown to 2-3 cm and then transferred to a 16hr-light/8hr-dark photoperiod (160 µmol/m^2^) at 28°C for a week before undergoing transformation.

### 2.2 *Agrobacterium* and vector materials

**Figure 1:**
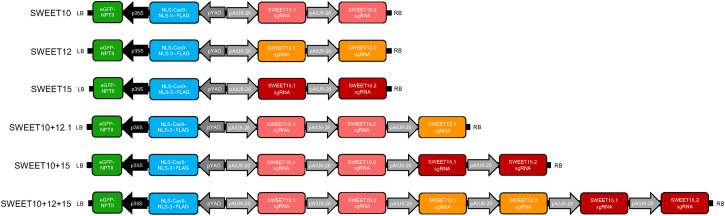
Schematic diagram of the constructs used for transformation. Two single guide RNAs (sgRNAs) were designed to target each of the *SWEET10*, *SWEET12* or *SWEET15* genes and each sgRNA was driven by an AtU6-26 promoter. The constructs include an eGFP-NPTII marker (eGFP, encoding enhanced green fluorescent protein; NPTII, encoding neomycin phosphotransferase) driven by the 35S promoter, encoding a fusion protein. Cas9 was driven by the YAO promoter.

CRISPR/Cas9 genome editing constructs were produced using a binary vector expressing Cas9 under the *Arabidopsis* YAO promoter as previously described (Zhang et al., 2017b). Briefly, the primers used to insert the different sgRNAs were synthesized and annealed, and then were ligated to an AtU6-26-sgRNA-SK vector. The AtU6-26 promoter with gRNA was then transferred to the proYAO-Cas9-NOS binary vector. For multiplex genome editing, additional AtU6-26-gRNA cassettes were added to the final vector at the *Spe*I site. **Figure 1** shows constructs used in this study. Constructs targeting *SWEET10*, *SWEET12* and *SWEET15* contain two sgRNA targets each. The double mutant construct SWEET10 + 12.1 consists of two sgRNAs targeting *SWEET10* and one sgRNA targeting *SWEET12* while SWEET10 + 15 consists of two sgRNAs targeting *SWEET10* and two targeting *SWEET15*. Similarly, the triple mutant construct SWEET10 + 12 + 15 consists of two sgRNAs targeting each of the three genes, *SWEET10*, *SWEET12* and *SWEET15.* Transgenic plants were selected by kanamycin (NPTII-neomycin phosphotransferase II) and GFP signal as a visual marker.

For transformation, *Agrobacterium* was streaked on a plate containing 25 mL YEP media with rifamcin (50 mg/L) and kanamycin (50 mg/L) and incubated in the dark overnight. A single colony was picked, plated on a new plate, and cultured for 24hr. *In planta* liquid medium containing 4.4 g/L MT (Murashige and Tucker) with vitamins, 40 g/L sucrose, 1.5 g/L glutamine, and 0.5 g/L malt extract, with pH adjusted to 5.8, was used to wash down the colonies into a 50 mL conical tube. A 1:1000 dilution of 0.1 M acetosyringone was added to the tube, which was then shaken at 180 rpm at 28°C for 1.5 to 2hr. The final OD_600_ was adjusted to 0.6-0.8 for transformation. For mock experiments, no *Agrobacterium* was added to the inoculation solution.

### 2.3 Assessing variation in regeneration and transformation efficiency due to *Agrobacterium* application techniques

**Figure 2:**
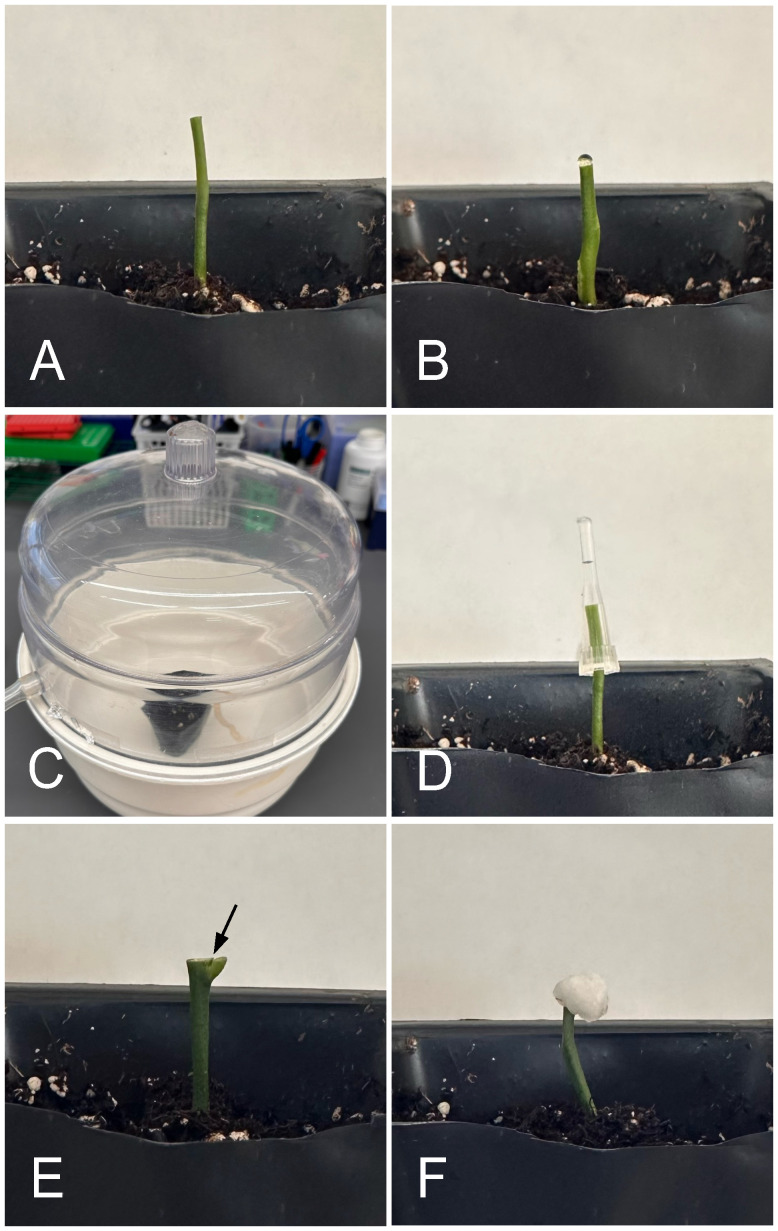
Different *Agrobacterium* application techniques. **(A)** Blunt-cut epicotyl for droplet, vacuum, and pipet tip inoculation. **(B)** Blunt cut with droplet inoculation (BCDI). **(C)** Blunt cut with droplet inoculation and vacuum infiltration (BCVI). **(D)** Blunt cut with pipet tip inoculation (BCTI). **(E)** Apical bud incision, arrow showing point at which axillary buds have been wounded. **(F)** Cotton inoculation (AGWC).

To identify the best *Agrobacterium* application techniques for shoot regeneration and recovery of transformed plants, we carried out the following inoculation techniques: blunt cut with vacuum infiltration (BCVI), blunt cut with tip inoculation (BCTI), blunt cut with droplet inoculation (BCDI), apical bud incision with micro wounds on axillary meristems followed by tip inoculation (AWT), apical bud incision with axillary meristems allowed to grow for 3-5 days and fresh micro wounds made followed by tip inoculation (AGWT), and apical bud incision with axillary meristems allowed to grow for 3-5 days and fresh micro wounds made followed by cotton inoculation (AGWC) (**Figures 2A–F**; **Table 1**). Detailed protocols for each method are presented below.

**Table 1:** Different *Agrobacterium* application techniques.

Method	Cut type	Inoculation method	Application duration
**BCVI**	Blunt cut	Vacuum infiltration	2min
**BCTI**	Blunt cut	Tip	1hr
**BCDI**	Blunt cut	Droplet	1hr
**AWT**	Apical bud cut, wounds on axillary meristems	Tip	24hr
**AGWT**	Apical bud cut, growth, wounds on axillary meristems	Tip	24hr
**AGWC**	Apical bud cut, growth, wounds on axillary meristems	Cotton	24hr

#### 2.3.1 Blunt cut with vacuum infiltration

Using a fresh blade, shoots including the apical bud and true leaves were cut off, leaving 3-4 cm of epicotyls (**Figure 2A**). A drop of *Agrobacterium* inoculation solution (10 µl) was added to the freshly cut epicotyls (**Figure 2B**). The seedlings were then placed in a vacuum chamber to allow infiltration for 2min (**Figure 2C**). After infection, the seedlings were removed from the vacuum chamber, and the wounds were tightly wrapped with parafilm. High humidity was maintained by wrapping the pots with plastic wrap. Inoculated seedlings were incubated in dark by covering with black plastic bags and placed in two different growth conditions for three days of co-culture: growth chambers at 28°C with 95% relative humidity (RH) and greenhouse at 25°C at 65%-75% RH. No watering was done during co-culture. After co-culture, seedlings were watered once every three days. The parafilm wrapping the wounds were then removed, and a cotton bud saturated with 50 mg/L kanamycin was used thrice to soak the wounds of the inoculated seedlings. After each kanamycin treatment, the cut tips were allowed to dry before the next soaking. The wounds were again re-covered with parafilm, and the pots were wrapped with plastic wrap before dark incubation. After two weeks of dark incubation from the day of infection, black plastic bags were removed, and the plants were grown in light (~160 µmol/m^2^ light intensity) at 28°C. Any new shoots growing from the cut site were screened for GFP signal using the NIGHTSEA system with royal blue LED light. All GFP-negative shoots were removed every 14 days, allowing GFP-positive shoots to grow.

#### 2.3.2 Blunt cut with tip inoculation

Freshly cut epicotyls were covered with a small pipet tip (10 µl) filled with *Agrobacterium* inoculation solution and incubated at room temperature for 1hr (**Figure 2D**). After infection, seedlings were wrapped with parafilm and co-cultured for three days in dark. Bagging of pots, growth in different environmental conditions, dark incubation and kanamycin treatments were done as described above.

#### 2.3.3 Blunt cut with droplet inoculation

A drop of *Agrobacterium* inoculation solution was added to the freshly cut epicotyls and plants incubated at room temperature for 1hr as shown in **Figure 2B**. After infection, seedlings were wrapped with parafilm and co-cultured for three days in dark. Bagging of pots, growth in different environmental conditions, dark incubation and kanamycin treatments were done as described above.

#### 2.3.4 Apical bud incision with wounds on axillary meristems followed by tip inoculation

Using a fresh blade, just the apical bud including true leaves were removed leaving 3-4 cm of epicotyls with axillary meristems. Three wounds on each meristem were made using a fine needle followed by tip inoculation for 24hr (**Figure 2E**) and all the subsequent steps including kanamycin treatment were performed as described above.

#### 2.3.5 Apical bud incision with axillary meristems grown for 3-5 days and fresh micro wounds made followed by tip inoculation

Using a fresh blade, just the apical bud including true leaves were removed leaving 3-4 cm of epicotyls with axillary meristems. The meristems were allowed to actively grow for 3-5 days. Following this, on the day of infection, a fresh cut on the tip of the epicotyl and three wounds on each meristem were made using a fine needle followed by tip inoculation for 24hr. Bagging of pots, growth in different environmental conditions, dark incubation and kanamycin treatments were done as described above.

#### 2.3.6 Apical bud incision with axillary meristems grown for 3-5 days and fresh micro wounds made followed by cotton inoculation

Using a fresh blade, just the apical bud including true leaves were removed leaving 3-4 cm of epicotyls with axillary meristems. The meristems were allowed to actively grow for 3-5 days. Following this, on the day of infection, when the axillary meristems are actively growing, a fresh crosscut on the tip of the epicotyl and three wounds on each meristem were made using a fine needle for *Agrobacterium* penetration. Epicotyls with fresh wounds were immediately covered with small cotton balls saturated with *Agrobacterium* inoculation solution and incubated for 1-2 days (**Figure 2F**). After incubation, cotton balls were removed, and the epicotyls were wrapped tightly with the parafilm. After three days of co-cultivation, the parafilm wrapping the wounds were then removed, and cotton balls saturated with 50 mg/L kanamycin was used thrice to soak the wounds of the inoculated seedlings. After each kanamycin treatment, the cut tips were allowed to dry before next soaking. The wounds were again re-covered with parafilm, and the pots were wrapped with plastic wrap before dark incubation. After two weeks of dark incubation from the day of infection, black plastic bags were removed, and the plants were grown in light (~160 µmol/m^2^ light intensity) at 28°C. Any new shoots growing from the cut site were screened for GFP signal using the NIGHTSEA system with royal blue LED light. All GFP-negative shoots were removed every 14 days, allowing GFP-positive shoots to grow.

### 2.4 Assessing variation in regeneration efficiency due to environmental conditions and seedling age

Our preliminary trials showed that high humidity is of utmost importance for plant regeneration and survival of the seedlings after infection. Therefore, we tested all the above-mentioned approaches in different environmental conditions to evaluate variation. The experiments were carried out in three different growth conditions: in growth chambers inside a black plastic bag (~95% RH), in capped glass test tubes (100% RH), and in a greenhouse inside a black plastic bag (65%-75% RH). Variation due to seedling age was evaluated by comparing regeneration efficiency of seedlings that were 4-6 weeks and 12+ weeks old for all the experiments listed above.

### 2.5 Heat stress and confirmation of transgene integration

GPF-positive shoots grown for 1.5-2 months were exposed to 14 cycles of heat stress treatments consisting of 72hr at 37°C and recovery for 24hr at 25°C. After heat treatment, genomic DNA was isolated from regenerated leaves as described previously with minor modifications (LeBlanc et al., 2018). Briefly, 50 mg of leaf tissue was ground in 500 µl of Extraction Buffer (200 mM of Tris-HCl pH 8.0, 250 mM NaCl, 25 mM ethylenediaminetetraacetic acid (EDTA) and 1% SDS). The tubes were vortexed, followed by centrifugation for 10 min at 3200xg. The supernatant was transferred to a new tube and 70 µl of isopropanol was added, followed by centrifugation for 10min at 3200xg. The supernatant was removed, DNA pellets were washed twice with 70% ethanol, and resuspended in 100 µl water. PCR was performed to confirm the editing events in the transformed lemon plants using the primers specific for the gRNAs listed in **Table 2**. Sequences obtained from Sanger sequencing were analyzed using Benchling for confirmation of gene editing. Knock out scores were calculated using Synthego ICE (v3.0) Analysis tool.

**Table 2:** Primers and guide RNAs (gRNAs) used in this study.

Gene	Guide name	Primer name	Primer sequence (5’-3’)	Amplicon size
***SWEET10***	gRNA1- TACAAGAAGAAATCAACGGA	SWT10-F	GAGAGAGAGAGTGATCTTAGCAGT	308 bp
	gRNA2- AGATGGCCATTCACCACTCT	SWT10-R	GCAATGTAGATGGTCTGCATGA	
***SWEET12***	gRNA1- TGCAAGAAGAAATCAACAGA	SWT12-F	ACTCATGATCCCTCGGTTTTTG	701 bp
	gRNA2- CTCTGTTCAGTGCAATGCTT	SWT12-R	CAGCCTCAAAGTGTACAACTGT	
***SWEET15***	gRNA1- TAATGTTCGATCACCATCAA	SWT15-F	GGTGCTTGGCCTCTTTTAAGAA	323 bp
	gRNA2- TATCTCGTTCCTCGTGTACC	SWT15-R	CGCATAGTAGAACCACAGCATT	

### 2.6 Statistical analyses

All the data were first analyzed for normality using the Shapiro-Wilk test. Normal data from variation in regeneration rate due to *Agrobacterium* application technique and regeneration rate using AGWC in different cultivars were analyzed by one-way analysis of variance (ANOVA) and significance levels were calculated by Tukey’s honest significance test, with p <0.05 considered significant. Similarly, data from variation in regeneration efficiency due to environmental conditions and seedling ages were analyzed using Student’s t test with p <0.05 considered significant. Error bars in the graph show standard deviation and the number of independent experiments is noted in the figure legends.

## 3 Results

### 3.1 Influence of *Agrobacterium* application techniques on shoot regeneration in different citrus cultivars

**Figure 3:**
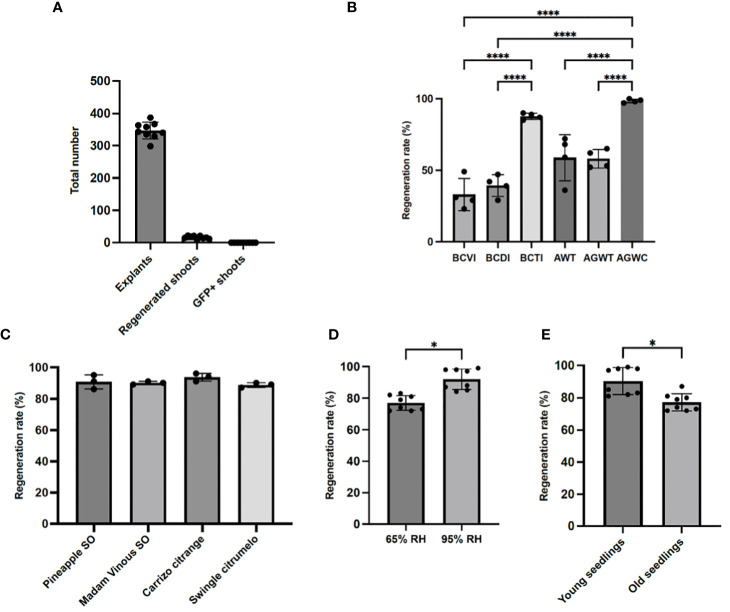
Regeneration rates under different parameters. **(A)** Numbers of plants regenerated and transformed in lemon using *in vitro* transformation in nine separate experiments. **(B)** Regeneration rate in lemon based on the methods of inoculation under different parameters; BCVI, blunt cut with vacuum infiltration; BCDI, blunt cut with droplet inoculation; BCTI, blunt cut with tip inoculation; AWT, Apical bud incision followed by micro mounds on axillary meristems and tip inoculation; AGWT, Axillary meristems grown for 3-5 days after apical bud incision followed by micro mounds on axillary meristems and tip inoculation; AGWC, Axillary meristems grown for 3-5 days after apical bud incision followed by micro mounds on axillary meristems and cotton inoculation. **(C)** AGWC protocol applied in different cultivars of interest. **(D)** Effects of relative humidity (RH) on regeneration rate in lemon using AGWC. **(E)** Effects of seedling age on regeneration rate in lemon using AGWC; young seedlings 4-6 weeks old, old seedlings 10-12 weeks old. Data were analyzed for normality using the Shapiro-Wilk test. In **(B, C)**, normal data were then analyzed by one-way analysis of variance (ANOVA) and significance levels were calculated by Tukey’s honest significance test, with p <0.05 considered significant. In B, four independent experiments were performed for each method and in **(C)**, three independent experiments were performed for each cultivar. In **(D, E)**, Student’s t test was used for statistical analyses, with p <0.05 considered significant. Eight independent experiments were performed for each group. Error bars in the graph show standard deviation and * indicates P<0.05 and **** indicates P<0.0001.

Both the regeneration efficiency and the effectiveness of a transformation method vary considerably among different citrus cultivars. Unlike Carrizo citrange (Pena et al., 1997; Dutt and Grosser, 2009), lemons are highly recalcitrant to *in vitro Agrobacterium*-mediated transformation (Dutt et al., 2009). Initially, we performed *Agrobacterium*-mediated *in vitro* transformation in lemon using epicotyls as explants. Out of 3120 explants, 149 shoots were recovered with a regeneration rate (number of shoots produced/total explants) of 4.8%. Moreover, none of the lemons we recovered exhibited GFP signal, indicating that none of the plants were transformed with the transformation rate (GFP-positive shoot/total explants) being 0% (**Figure 3A**; **Table 3**). This whole process of *in vitro* tissue culture took several months and was labor intensive. In search of an alternative approach that bypasses the laborious tissue culture steps, we developed and optimized an *in planta* transformation method in citrus. Using lemon, we examined regeneration efficiency in response to different application techniques to define the best method for *Agrobacterium* inoculation. **Table 1** shows different *Agrobacterium* application methods that were assessed. We identified AWGC to be the best technique to inoculate the plants and achieve maximum regeneration as well as prevent dying of the cut seedlings at the same time, followed by BCTI. Seedlings inoculated with *Agrobacterium* using the AWGC technique had a higher level of shoot regeneration which aided in the recovery of transformed shoots as compared to the other techniques (**Figure 3B**). The regeneration rate (number of shoots produced/total seedlings) varied greatly between the different techniques. Regeneration rates under different conditions ranged between 23.33%-48.33% for BCVI, 28.33%-46.67% for BCDI, 36.67%-71.67% for AWT, and 51.67%-65% for AGWT (**Figure 3B**; **Supplementary Table 1**). Both AGWC and BCTI had high regeneration rates of over 95% and 85% respectively, but the latter took longer time (4-5 months as compared to 2-3 months in AGWC) to produce shoots in all the seedlings (data not shown). Overall, all the different application methods that were tested showed higher regeneration efficiency in lemon compared to 4.8% in *in vitro* transformation (**Figures 3A, B**). Remarkably, the regeneration efficiency using the AGWC method of transformation was over 95% in lemon and over 85% in other varieties used in this study including Pineapple sweet orange (**Figures 3B, C**; **Supplementary Table 2**). Hence, *in planta* transformation using AGWC for *Agrobacterium* application was identified to be the best technique for maximum shoot regeneration.

**Table 3:** Transformation efficiency using AGWC and BCTI.

Inoculation method	Cultivars	Construct used	# of seedlings	# of GFP+ shoots	Transformation efficiency (%)
***In vitro***	Lemon	SWEET10+12+15	3120	0	0%
**AGWC**	Lemon	SWEET10+12+15	440	5	1.14%
**AGWC**	Lemon (Mosaics)	SWEET10+12+15	440	2	0.45%
**BCTI**	Lemon	SWEET10	24	1	4.17%
**BCTI**	Lemon	SWEET12	106	1	0.94%
**BCTI**	Lemon	SWEET15	120	1	0.83%
**BCTI**	Lemon	SWEET10+12.1	160	1	0.63%
**BCTI**	Lemon	SWEET10+15	75	1	1.33%
**BCTI**	Lemon	SWEET10+12+15	89	1	1.12%
**AGWC**	Pineapple sweet orange	SWEET10+12+15	360	3	0.83%
**AGWC**	Carrizo citrange	SWEET10+12+15	74	3	4.05%

### 3.2 Influence of growth condition and seedling age on shoot regeneration

It has been established that a dark incubation period is critical for shoot regeneration in citrus (Marutani-Hert et al., 2012; Zhang et al., 2017a). To this end, all the different growth conditions that were tested, were incubated in dark at 28°C for 2 weeks to promote shoot regeneration. To test the importance of different environmental factors on shoot regeneration, we first decided to test the effect of relative humidity on the rate of shoot regeneration. Three different environmental conditions were tested: growth chambers inside a black plastic bag with ~95% RH, capped glass test tubes with 100% RH, and greenhouse inside a black plastic bag 65%-75% RH. In capped glass tubes, seedlings exposed to 100% relative humidity consistently displayed significant contamination, precluding further analyses (data not shown). Our results showed that seedlings incubated in a growth chamber inside a black plastic bag for two weeks of dark incubation with 95% RH had an average of 92% shoot regeneration rate as compared to 77% in seedlings grown in greenhouse condition with 65% RH (**Figure 3D**). In conclusion, a high relative humidity in addition to dark incubation is also needed to increase shoot regeneration.

**Figure 4:**
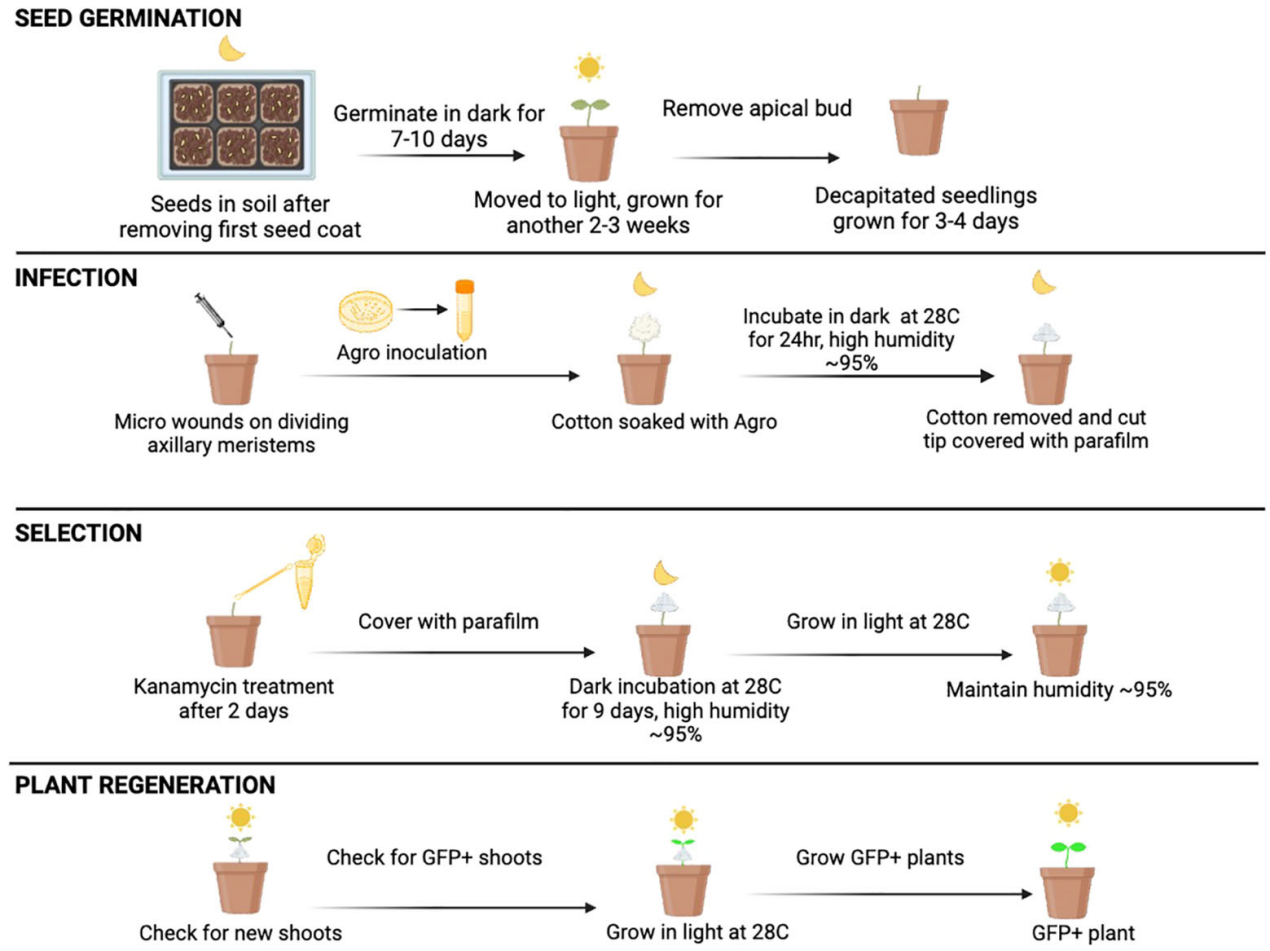
Optimized *Agrobacterium*-mediated in planta transformation using AGWC in Limoneira 8A Lisbon Lemon. Decapitated (apical bud removed) four to six weeks old seedlings grown for three to four days. Fresh micro-wounds made using a fine needle when axillary meristems were actively dividing. Wounds covered with cotton balls saturated with inoculation solution containing Agrobacteria and incubated for 1 day in dark inside thick black plastic bag. Cotton balls removed the next day, and wounds wrapped with Parafilm and incubated in dark again. Selection with kanamycin on day five after first inoculation. Dark incubation for a total of two weeks from day 1. Black plastic bag removed, and seedlings grown in light after two weeks. Humidity maintained at 90-95% throughout the process. GFP negative shoots peeled every two weeks. Any GFP positive shoots moved to a new pot and grown for further evaluation.

Next, we tested the influence of the age of seedlings on shoot regeneration by comparing the shoot regeneration in 4-6 weeks-old seedlings and 12+ week-old seedlings using AGWC method of *Agrobacterium* application. Our results show that 4-6 weeks old seedlings regenerated significantly more shoots compared to 12+ weeks old seedlings, 90% and 76% respectively (**Figure 3E**). All together our data indicate that high relative humidity and young seedlings are favorable to allow shoot regeneration in citrus. **Figure 4** shows the overall optimized AGWC protocol that helped in maximum recovery of transformed shoots. BCTI is a similar protocol except for seedling cut type and *Agrobacterium* inoculation step.

### 3.3 Efficiency of *in planta* transformation in citrus

**Figure 5:**
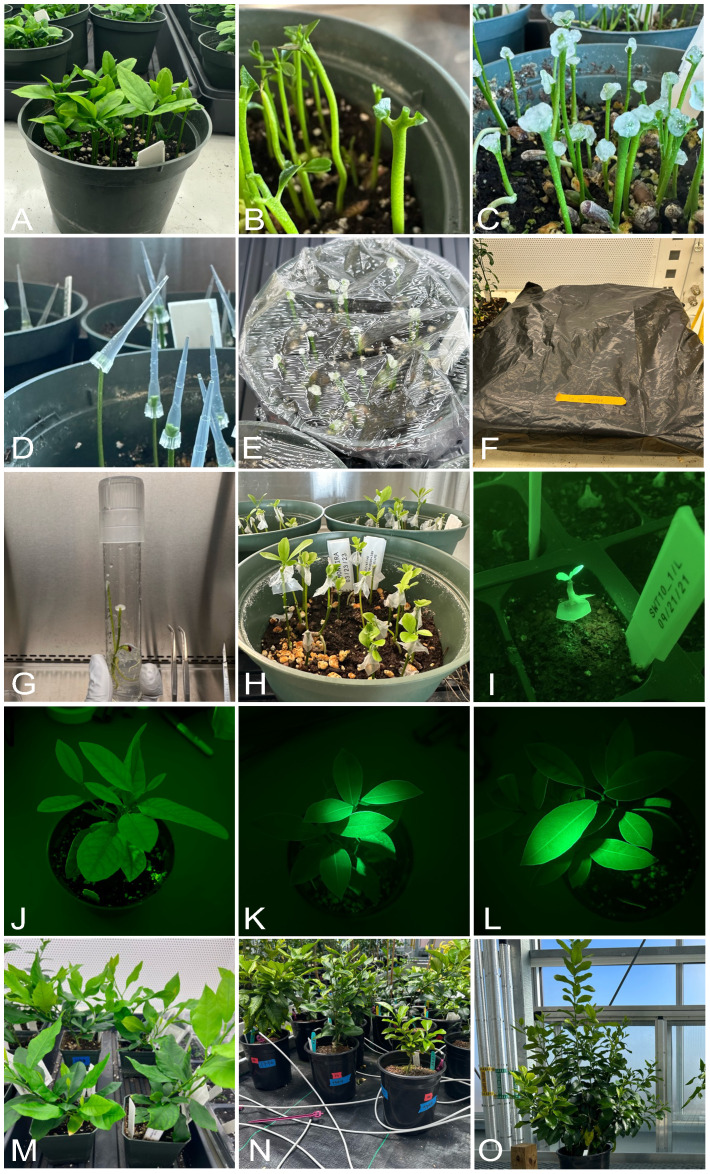
*In planta* transformation in lemon. **(A)** young seedlings between five to six weeks used for transformation. **(B)** apical bud removed, and seedlings grown for three to four days. **(C)** *Agrobacterium* application using cotton balls after making fresh wounds on the rapidly dividing axillary meristems. **(D)** *Agrobacterium* application using tip inoculation. **(E)** high humidity maintained by wrapping the pots with saran wrap. **(F)** two weeks of dark incubation inside thick black plastic bags. **(G)** In glass *in planta* transformation using cotton inoculation. **(H)** regenerated shoots after two-three months. **(I)** identification of transformed plants by checking for GFP signal. **(J)** wild type lemon without GFP signal. **(K, L)** transgenic lemon showing GFP signals. **(M–O)** transgenic plants grown in growth chambers and greenhouse.

Based on the above results, *in planta* transformation protocols, AGWC and BCTI under high relative humidity of around 95% and using young seedlings between the age of 4-6 weeks was found to be the most effective for increasing shoot regeneration rate, which is an essential requirement for *Agrobacterium*-mediated transformation experiments. The *in planta* transformation method developed in this study produced 11 transgenic lines of lemon, and three transgenic lines of Pineapple sweet orange. **Table 3** shows the transformation efficiency in lemon and Pineapple sweet orange using the AGWC and BCTI methods. Using the AGWC method, we obtained five transgenic lines of lemon (1.14% transformation rate) from 440 seedlings and three transgenic lines of Pineapple sweet orange (0.83% transformation rate) from 360 seedlings, using the SWEET10 + 12 + 15 construct. Construct SWEET10 + 12 + 15 is a construct consisting of two sgRNAs targeting each of three genes, *SWEET10*, *SWEET12* and *SWEET15.* Additionally, the BCTI method produced six transgenic lines of lemon using different constructs (**Table 3**), with transformation rates as follow: 4.17% (SWEET10), 0.94% (SWEET12), 0.83% (SWEET15), 0.63% (SWEET10 + 12.1), 1.13% (SWEET10 + 15), and 1.12% (SWEET10 + 12 + 15). Two mosaic plants were also obtained using the AGWC method, indicating a multicellular origin of regeneration buds. Moreover, we also obtained three transgenic lines of Carrizo citrange using AGWC method with the SWEET10 + 12 + 15 construct, out of 74 seedlings with the transformation rate of 4.05% (**Table 3**). Although the *in planta* transformation efficiency remains low in these varieties, it is a promising alternative to *in vitro* methods. **Figure 5** summarizes the *in planta* transformation process in citrus as a valuable alternative method to *in vitro* tissue-culture based transformation.

### 3.4 Genotyping, verification of CRISPR activity and gene editing efficiency

After two weeks of dark incubation, all the seedlings were transferred to a light condition. Within 4-6 weeks, the wounded site produced many new shoots. GFP fluorescence was used as a visual marker to identify transformed GFP-positive shoots. These shoots were grown for 1.5-2 months and then exposed to 14 cycles of heat stress (72hr at 37°C followed by 24hr recovery at 25°C) to enhance Cas9 activity and the mutagenesis rate by CRISPR/Cas9 (LeBlanc et al., 2018). PCR analysis and Sanger sequencing of the *SWEET10*, *SWEET12* and *SWEET15* genes were performed with leaf discs from each GFP-positive shoot to assess transgene integration and gene editing rates. Synthego ICE analysis confirmed mutations in the targeted genes and calculated knock out scores, with most seedlings showing high knockout scores (over 90%) for at least one of the two sgRNAs (**Table 4**). This confirms the successful gene editing of all the 14 transgenic lines obtained from the above mentioned *in planta* protocol. Mutations were also verified using Benchling (data not shown).

**Table 4:** List of 14 transformed lines with knock out score (KO Score) for each target gene. PSO stands for Pineapple sweet orange.

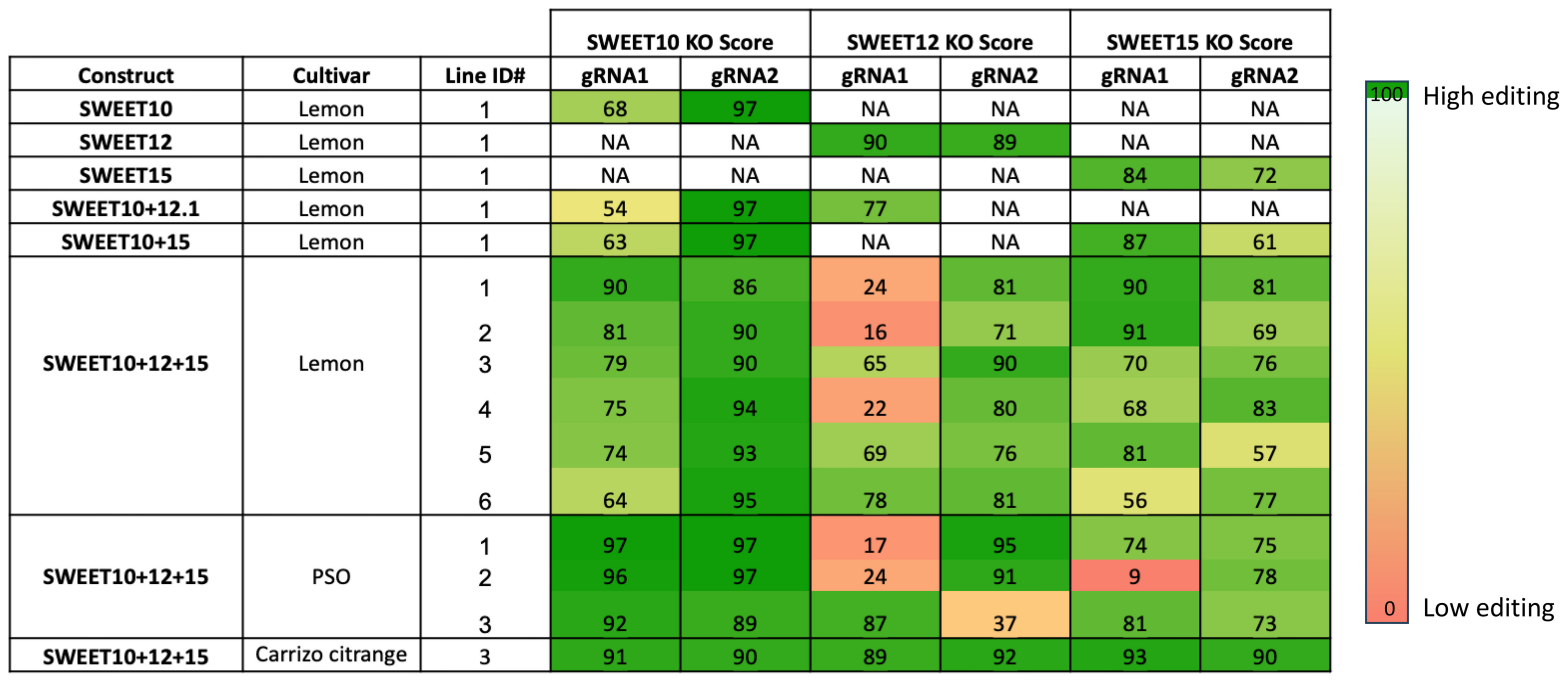

## 4 Discussion

In this study, we optimized the methodology for *in planta* transformation in citrus, producing transgenic lines in lemon and Pineapple sweet orange within 3-4 months, significantly faster than conventional approaches. Shoot regeneration is an essential prerequisite for *Agrobacterium*-mediated transformation. Therefore, it is important to identify factors that promote shoot regeneration. BCVI and BCDI methods promoted low shoot regeneration rates of 23.33%-48.33% and 28.33%-46.67%, respectively. AWT and AGWT methods also had moderate shoot regeneration rates, 36.67-71.67% and 51.67-65%, respectively. We suspect that the lower regeneration rates in these methods was likely due to wilting shoot tips and bacterial overgrowth at the cut site, which killed the seedlings. The AGWC method proved to be the best, with a shoot regeneration rate of over 95% in lemon and over 85% in other cultivars, within three months (**Figures 3B, C**). Unlike tip inoculation in AWT and AGWT where the cut part is exposed to inoculation solution for over 24hr, the cotton inoculation method did not lead to obvious bacterial overgrowth or tip wilting, as during the 24hr inoculation, moisture in the cotton is lost and the cotton balls eventually dries up, not submerging the cut sites in inoculation solution for a prolonged time but providing ample time for bacteria to penetrate the wounded site. BCTI also had a shoot regeneration rate of over 85%, but it was a slower process of four to five months as compared to two to three months in AGWC.

High humidity is crucial for shoot growth after inoculation. Out of the different environmental conditions tested, the best condition was dark incubation in growth chamber with 95% RH resulting in over 92% shoot regeneration and higher recovery of transformed shoots (**Figure 3D**). The result seen in this study corroborates the reports of Marutani-Hert et al. (2012), suggesting that two weeks of dark incubation is indispensable for citrus shoot regeneration. Seedling age also had a significant impact on the rate of shoot regeneration. Young seedlings between the age of 4-6 weeks had higher shoot regeneration rate compared to the older seedlings of 12+ weeks age (**Figure 3E**).

Most citrus species are recalcitrant to *Agrobacterium*-mediated transformation, hampering the development of novel citrus varieties through genetic engineering (Singh and Rajam, 2010). Regeneration and transformation efficiencies vary in different cultivars. Some cultivars are more responsive to transformation such as citranges (Omar et al., 2016; Dutt et al., 2018) and Duncan grapefruit (Dutt and Grosser, 2009; Orbović and Grosser, 2015), while others, such as Clementine (Cervera et al., 2008), sour orange (Dutt and Grosser, 2009) and lemons (Dutt et al., 2009), are highly recalcitrant to both regeneration and *Agrobacterium*-mediated *in vitro* transformation (**Figure 3A**). Here we show that the *in planta* transformation method significantly improved regeneration and transformation rates in lemon compared to *in vitro* methods, increasing the regeneration rate from 4.58% to over 95%, and the transformation rate from 0 to up to 4.17%.

Using the AGWC method, we obtained a total of five transgenic lines of lemon, and two mosaic lemon lines. Similarly, with the BCTI method, we obtained a total of six transgenic lines of lemon with the transformation efficiency ranging between 0.63% and 4.17% for different constructs (**Table 3**). In addition, we also obtained three transgenic lines of Pineapple sweet orange and three transgenic lines of Carrizo citrange using the AGWC method of inoculation. It must be noted that, the optimization of *in planta* transformation in this study is mainly for recalcitrant citrus varieties. *In vitro* transformation in Carrizo citrange has a very high regeneration and transformation rate (Omar et al., 2016; Dutt et al., 2018) as the explants produce calli which in turn produce multiple new shoots. The transformation rate in Carrizo citrange using *in planta* transformation was only 4.05% as the seedlings do not form calli at the cut site and we rarely obtain multiple transformed shoots per seedling unlike in *in vitro* approach. The regeneration rates for Madam Vinous sweet orange and Swingle citrumelo were over 86.67% but they failed to produce any transformed shoots.

The high occurrence of escapes (non-transformed plants) in our experiments can be attributed to inefficient selection, likely due to the protection of non-transformed cells (Ghorbel et al., 1999). The adoption of selectable marker genes is a standard practice in genetic transformation technology for the effective retrieval of transgenic plants. Implementation of efficient selection strategies heightens the occurrence of transgenic events, thereby leading to greater probability of regenerating transgenic plants. Typically, selection is often based on the resistance to antibiotics and herbicides (Ballester et al., 2008). Kanamycin ranks among the top selective antibiotics employed in transformation processes. In this study, we used kanamycin as selection agent. Cotton balls saturated with 50 mg/L kanamycin were used to soak the cut epicotyl tips thrice, allowing cut tips to dry between each application similar to the selection done in *in planta* transformation of pomelo (Zhang et al., 2017a). In our initial experiments, we performed two separate applications of kanamycin for selection; three days post inoculation and two days post first kanamycin treatment, but it led to burnt-appearing epicotyl tips and did not produce any shoots likely because of kanamycin sensitivity (data not shown). Hence transformation efficiencies in our study were calculated based on a single kanamycin application for selection.

Despite the relatively low transformation efficiency in lemon and Pineapple sweet orange, we generated these transgenic lines in less than three months using AGWC method and in less than six months using BCTI method. Hence the best method for *in planta* transformation in citrus is AGWC followed by BCTI. In AGWC, axillary meristems are grown for 3-5 days after apical bud removal and then injured using a fine needle when the meristems are rapidly growing such that actively dividing cells are infected with *Agrobacterium*. BCTI, on the other hand is a simple blunt cut of the epicotyl where new shoots are produced from the adventitious buds. Even though both the methods showed very high regeneration rates of over 85%, recovery of both transformed and non-transformed shoots was faster in AGWC as compared to BCTI method. AGWC being a rapid and efficient method as compared to BCTI both in terms of regeneration and transformation could be attributed to the fact that actively multiplying cells in axillary meristems are targeted in AGWC methods, producing shoots faster than in the case of BCTI where the shoots are regenerated from the adventitious buds. This rapid timeline contrasts with the conventional *in vitro* approach, bypassing laborious tissue culture steps. Furthermore, our outlined approaches are cost-efficient and easy to implement, making an *in planta* approach to citrus transformation appealing for researchers interested in the genetic improvement of citrus.

## 5 Conclusion

In this study, a methodology for *Agrobacterium*-mediated *in planta* transformation in citrus was optimized, overcoming limitations of conventional transformation approaches. This protocol offers a new tool that combines improved regeneration efficiency with successful transformation, while reducing time, labor, and production costs. The optimized *in planta* transformation protocol in the present study will be useful for the genetic improvement of citrus, including engineering for disease resistance, fruit quality, and other desirable characteristics.

## Data availability statement

The datasets presented in this study can be found in online repositories. The names of the repository/repositories and accession number(s) can be found in the article/**Supplementary Material**.

## Author contributions

AK: Methodology, Visualization, Formal analysis, Investigation, Writing – original draft. CS: Investigation, Methodology, Writing – review & editing. CV: Methodology, Writing – review & editing. BM: Methodology, Writing – review & editing. VI: Methodology, Writing – review & editing, Conceptualization, Funding acquisition, Project administration, Supervision, Visualization.
