# Supplementary material for: Optimization of in planta methodology for genome editing and transformation in Citrus
Source: Front Plant Sci. 2024 Jul 12;15:1438031. doi: 10.3389/fpls.2024.1438031 (PMC11272483; doi:10.3389/fpls.2024.1438031)
Supplement: Supplementary file 1 [file DataSheet_1.pdf]

Supplementary Table 1. Shoot regeneration rate in lemon using different inoculation techniques.

Inoculation method	Construct used	Cultivar	# of seedlings used	# of regenerated shoot	Regeneration rate (%)
**BCVI**	SWEET10+12+15	Lemon	60	29	48.33%
**BCVI**	SWEET10+12+15	Lemon	60	19	31.67%
**BCVI**	SWEET10+12+15	Lemon	60	14	23.33%
**BCVI**	SWEET10+12+15	Lemon	60	17	28.33%
**BCDI**	SWEET10+12+15	Lemon	60	17	28.33%
**BCDI**	SWEET10+12+15	Lemon	60	23	38.33%
**BCDI**	SWEET10+12+15	Lemon	60	25	41.67%
**BCDI**	SWEET10+12+15	Lemon	60	28	46.67%
**AWT**	SWEET10+12+15	Lemon	60	41	68.33%
**AWT**	SWEET10+12+15	Lemon	60	35	58.33%
**AWT**	SWEET10+12+15	Lemon	60	43	71.67%
**AWT**	SWEET10+12+15	Lemon	60	22	36.67%
**AGWT**	SWEET10+12+15	Lemon	60	39	65%
**AGWT**	SWEET10+12+15	Lemon	60	34	56.67%
**AGWT**	SWEET10+12+15	Lemon	60	31	51.67%
**AGWT**	SWEET10+12+15	Lemon	60	37	61.67%

Supplementary Table 2. Shoot regeneration rate in different cultivars using AGWC method of inoculation.

Cultivar	Construct used	Inoculation method	# of seedlings used	# of regenerated shoot	Regeneration rate (%)
**Pineapple sweet orange**	SWEET10	AGWC	60	52	86.67%
**Pineapple sweet orange**	SWEET10	AGWC	60	57	95%
**Pineapple sweet orange**	SWEET10	AGWC	40	36	90%
**Carrizo citrange**	SWEET10+12+15	AGWC	26	24	92.30%
**Carrizo citrange**	SWEET10+12+15	AGWC	20	19	95%
**Carrizo citrange**	SWEET10+12+15	AGWC	28	26	92.85%
**Madam Vinous sweet orange**	SWEET10	AGWC	60	53	88.33%
**Madam Vinous sweet orange**	SWEET10	AGWC	60	55	91.67%
**Madam Vinous sweet orange**	SWEET10	AGWC	60	54	90%
**Swingle citrumelo**	SWEET10	AGWC	60	54	90%
**Swingle citrumelo**	SWEET10	AGWC	60	52	86.67%
**Swingle citrumelo**	SWEET10	AGWC	60	53	88.33%
